# Community perception and preventive practices regarding malaria in low-endemicity regions on Indonesian Kalimantan border adjacent to high-endemicity zoonotic malaria in Malaysian Borneo

**DOI:** 10.1186/s41182-025-00757-x

**Published:** 2025-05-23

**Authors:** Diana Natalia, Willy Handoko, Sari Rahmayanti, Tri Wahyudi, Khamisah A. Kadir, Zulkarnain Md Idris, Ayu A. A. Rashid, Paul C. S. Divis

**Affiliations:** 1 Malaria Research Centre, Faculty of Medicine and Health Sciences, Universiti Malaysia Sarawak, Kota Samarahan, Malaysia; 2 Faculty of Medicine, Universitas Tanjungpura, Pontianak, Indonesia; 3 Department of Parasitology and Medical Entomology, Faculty of Medicine, Universiti Kebangsaan Malaysia, Kuala Lumpur, Malaysia

**Keywords:** Knowledge, Attitude, Preventive practice, Malaria, Kalimantan

## Abstract

**Background:**

Indonesia aspires to completely eliminate malaria by 2030. Malaria cases have fallen drastically due to the implementation of national strategic plans and policies, and the Ministry of Health has granted certification of elimination status to various areas, including Kalimantan. However, this low prevalence contrasts sharply with the continued high prevalence (18.9%, totalling 3290 cases) of *Plasmodium knowlesi* infections in Malaysian Borneo. Assessing the knowledge and preventive practices regarding malaria and attitudes towards zoonotic malaria within communities along the Kalimantan border is essential to understanding the low endemicity (API < 1) of malaria in this region.

**Methods:**

Between February and April 2021, a structured questionnaire was administered to respondents who lived in villages with recent malaria cases (*P. vivax* and *P. falciparum* infections) across the West, East, and North Kalimantan provinces bordering Malaysian Borneo. The questionnaire collected demographic information, knowledge, prevention practices, illness management, and attitudes towards contributing factors of zoonotic malaria. Data were analysed using descriptive statistic and the association between variables was determined using logistic regression. A *P* value less than 0.05 was considered statistically significant.

**Results:**

Of the 639 respondents, 47.6% had completed primary education, and 49.1% worked in the agricultural sector. More than half of the respondents had good knowledge (58.2%) and good practice (51%) regarding malaria's cause, symptoms and prevention. A notable 58.9% could identify at least two classic symptoms of malaria (fever and shivering), and 78.6% associated the disease with mos quito bites. More than half of the respondents (53.7%) owned bed nets and stated using them every night on a regular basis (49.3%). However, more than half of these bed nets were not insecticide-treated. Indoor residual spraying by the health authority was uncommon. A common practice was that 84% of respondents sought treatment at health facilities when suspecting malaria (fever and shivering). Regarding the potential for acquiring zoonotic malaria, 36.2% of respondents lived near the forest, and 15.8% reported encountering monkeys within 500 m of their house. Multivariate analysis showed that an increase in education level significantly predicted good knowledge of malaria. Meanwhile, good malaria practices were significantly associated with women (aOR = 2.25; *P* < 0.001), age 25–64 (aOR = 2.64; *P* < 0.001), and age over 65 (aOR = 3.06; *P* = 0.004).

**Conclusions:**

This study observed an exceptional level of malaria awareness among these communities. However, it is crucial to emphasise the importance of continuous malaria surveillance within this community for maintaining the current low malaria cases and achieving the goal of malaria-free status in the country by 2030.

## Background

Malaria remains endemic in Indonesia, posing a significant threat to the well-being of its population, with an estimated 273 million individuals at risk [1]. Each year, several million cases of malaria are reported, caused by four known species of human *Plasmodium: P. falciparum, P. vivax, P. malariae,* and *P. ovale* [2, 3]. Notably, extensive malaria control efforts in Indonesia over the past two decades have led to a significant reduction in malaria morbidity and mortality, in line with trends observed in malaria-endemic Southeast Asian countries [1]. Malaria incidence has reduced by 83% from 18 cases per 1000 population at risk in 2000 to about three cases per 1000 population at risk in 2022 and malaria death dropped by 77% from 2000 to 2022 in WHO South–East Asia Region [1]. As evidence of progress in Indonesia, 72% of malaria-endemic regions have been granted malaria-free status [4].

Using molecular approaches, *Plasmodium* species can be identified with greater precision [5]. Molecular analysis has unveiled the prevalence of *Plasmodium knowlesi*, a simian *Plasmodium* species recognized as the fifth *Plasmodium* species that infects humans [6]. Following the discovery of high frequencies of *P. knowlesi* infections in Malaysia [7], all Southeast Asian countries, except Timor Leste, have reported *P. knowlesi* infection in humans [7–14]. Cases of *P. knowlesi* malaria were low in Indonesia, with reports mainly from Sumatera [15, 16] and a small number of cases in Kalimantan [17–20].

Besides *P. knowlesi*, six other simian *Plasmodium* species have been reported to be transmissible to humans by mosquito bite and cause malaria. These include *P. cynomolgi* [21–24], *P. brasilianum* [25, 26], *P. eylesi* [27, 28], *P. inui* [29], *P. schwetzi* [30], and *P. simium* [31]. Although long-tailed (*Macaca fascicularis*) and pig-tailed (*M. nemestrina*) macaques have been identified as the natural reservoir for most of those simian *Plasmodium* species from Southeast Asia [32], the natural hosts for these *Plasmodium* species are very diverse, which include Old World and New World primates [33, 34]. Other primates across Southeast Asia, such as banded leaf monkeys (*Presbytis melalophos*) [3], northern pig-tailed macaques (*M. leonina*) [35], stump-tailed macaques (*M. arctoides*) [36] and dusky leaf monkeys (*Semnopithecus obscurus*) [37], are also potential hosts.

The massive destruction of Kalimantan's rainforests continues largely unabated, endangering the local environment [38, 39]. The contact pattern between the human–vector–host is shaped by movement and land use in the environment [40, 41]. Factors such as oil palm plantations, seasonal flooding, and rampant bushfires, all stemming from deforestation exacerbate the ecological peril faced by the Kalimantan forest [33, 42]. These changes may have led to an increased presence of certain *Anopheles* species as vectors in agricultural areas and villages [43]. *An. latens*, incriminated as *P. knowlesi* vector in Kapit, Sarawak, was abundantly discovered in the forest region, also found in the farm near the forest and the village nearby [43]. A reduction in macaque populations could increase the frequency of mosquito bites per surviving macaques in the same region [44]. In Brazil, 48 autochthonous cases (human blood samples) formerly diagnosed as *P. vivax,* 28 of them are now believed to have been *P. simium* infections [45], which was previously considered a monkey-specific malaria parasite. It is possible that the vacant ecological niche in this region made *P. simium* more susceptible to a host switch back to humans [46]. Such behavioral shifts may enhance the prevalence of zoonotic malaria transmissions among the remaining macaque population, consequently increasing the risk of spillover infection in humans.

Economic disparities between Indonesian provinces and Malaysian states have led to cross-border migration on the Kalimantan–Malaysian Borneo border, impacting trade and infectious disease dynamics. While economic opportunities draw Indonesians to Sarawak and Sabah states in Malaysia, the movement of natural hosts such as wild macaques raise concerns about malaria parasite transmission in the border area. Zoonotic malaria caused by *P. knowlesi* is a particularly concerning infectious disease in Malaysian Borneo, posing new challenges for healthcare systems and public health efforts in Indonesia [1, 47].

Therefore, this study aimed to assess the level of awareness knowledge, preventive practices on malaria and attitudes towards zoonotic malaria in the low-endemicity area of Kalimantan, Indonesia, which shares a border with the high-endemicity zoonotic malaria area in Malaysian Borneo. Understanding the awareness and behaviors of local communities is essential for identifying and addressing the gap between awareness and action, thereby enhancing malaria prevention initiatives in the affected areas.

## Materials and methods

### Ethics statement

This study was approved by the Universitas Tanjungpura Ethics Committee (No 6639/UN22.9/TA.00.03/2019). Respondents were provided with a patient information sheet, outlining the topic, purpose, and benefits of the study, as well as ensuring the absolute confidentiality of the information obtained. Written consent was obtained before data collection, and respondents were informed of their right to withdraw from the study at any time. For respondents under the age of 17, informed consent was obtained by their guardians or parents; since some heads of household hesitated to be interviewed, they granted permission to their children to answer the questions. To avoid courtesy bias and improve self-report accuracy, interviews were done by the primary researcher, visiting each respondent’s residence, explaining each question in the questionnaire and interview procedures with face-to-face interviews in Bahasa Indonesia. All data collected were anonymized and securely stored with restricted access, and personal identifiers were removed to protect the privacy and confidentiality of the participants. We also obtained verbal consent from local community leaders to ensure the study approach was culturally appropriate and respectful.

### Study sites

Indonesia utilises the Annual Parasite Incidence (API) indicator as a key metric for assessing malaria morbidity. This metric is derived by dividing the total number of individuals positive for malaria (confirmed cases of malaria by microscopy or rapid diagnostic test) [48] by the population at risk within a given regency or province. Between 2015 and 2020, Indonesia successfully maintained the API indicator to below 1. However, in 2021, Indonesia experienced an API resurgence, reaching 1.1 and continued to rise to 1.6 in 2022 and 1.5 in 2023 [49].

Nearly all regencies within Kalimantan provinces have been declared as having low malaria endemic status (API < 1). Notably, East Kalimantan Province recorded the highest malaria morbidity at API 0.9, followed by North Kalimantan (API 0.2), South Kalimantan (API 0.1), Central Kalimantan (API 0.1) and West Kalimantan (API < 0.1) [49].

**Fig. 1:**
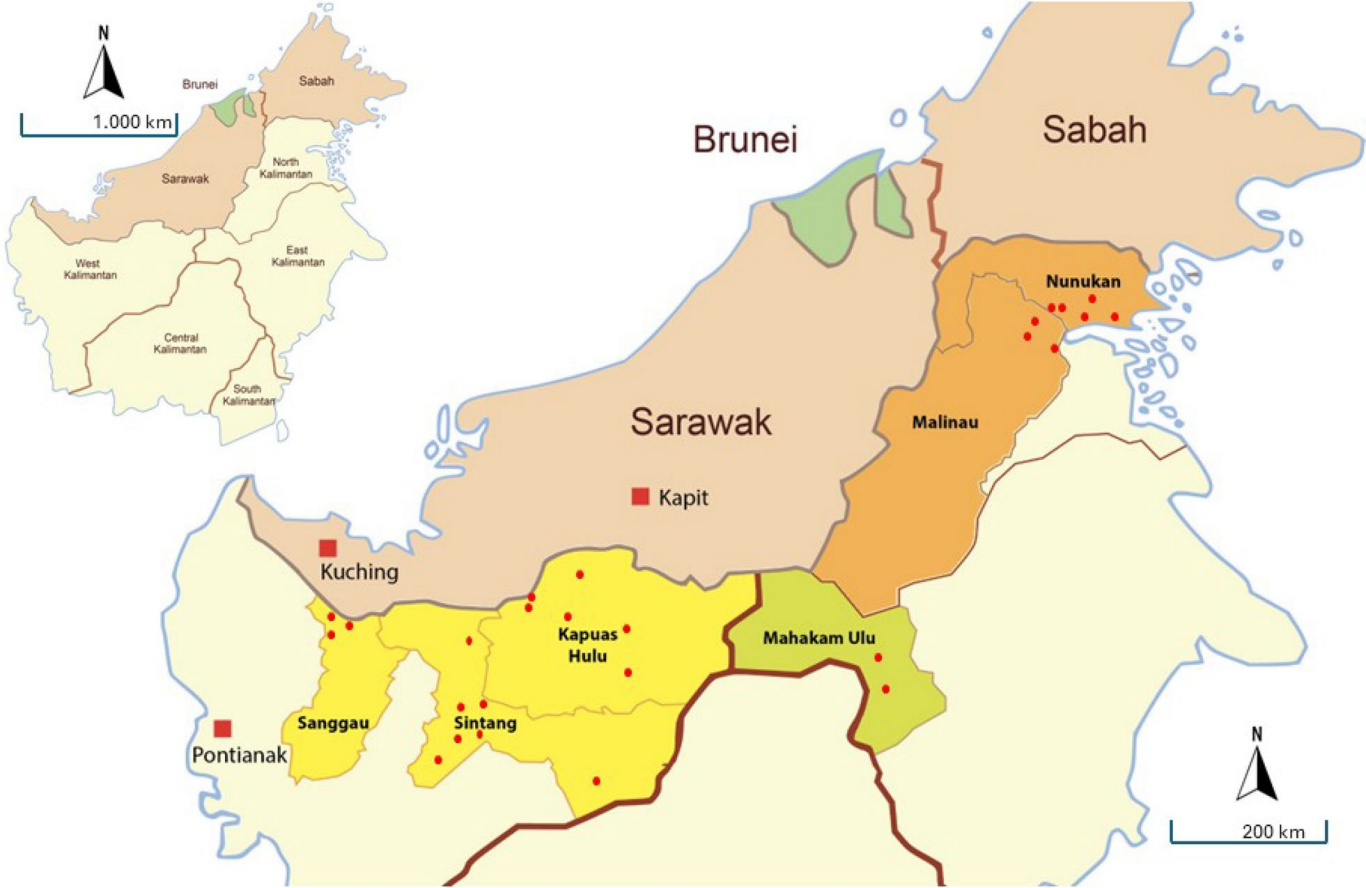
Map of the study sites. The map on the left shows Borneo Island which consists of three bordering countries—Brunei, Malaysia (Sarawak and Sabah states) and Indonesia (five Kalimantan provinces). The map on the right shows six regencies bordering Borneo Malaysia: Sanggau, Sintang and Kapuas Hulu Regency of West Kalimantan; Mahakam Ulu Regency of East Kalimantan; Malinau and Nunukan Regency of North Kalimantan, where the study was conducted. The red dots represent the surveillance that was conducted (Source: Google Map)

This study encompassed six regencies of Kalimantan, Indonesia, which share a border with Borneo, Malaysia (Fig. 1): Sanggau, Sintang, and Kapuas Hulu regencies of West Kalimantan Province; Malinau and Nunukan regencies of North Kalimantan Province; and Mahakam Ulu Regency of East Kalimantan Province. Kapuas Hulu Regency, located in the eastern region of West Kalimantan, is characterized by fewer settlements and more forests. Much of the natural forest in this province has been modified and developed, primarily for palm plantations, traditional agricultural fields, and human settlements, with farming as the main source of income. Danau Sentarum, a conservation forest within Kapuas Hulu regency, is home to various wildlife, including monkeys.

Mahakam Ulu is the only regency in East Kalimantan Province that shares an international border with Sarawak, Malaysia. Natural forests and numerous rivers predominantly cover this regency. It can only be reached by land or along the Mahakam River, requiring approximately 13 h of travel from Samarinda City, the capital of East Kalimantan Province. The majority of the population is Dayak, and their main sources of income are farming and hunting in the forest.

North Kalimantan Province, consisting of Malinau and Nunukan regencies, has extensive road access and is largely dominated by palm oil plantations. The main ethnicities in the area are Dayak and Malay, with Buginese migrant workers from the eastern region of Indonesia. Common occupations include farming, hunting, and plantation work. People often use small boats to cross the Snake River (*Sungai Ular*) in Simanggaris of Nunukan regency, for trade products or ferry boats from Tarakan, Indonesia, to Tawau town of Sabah, Malaysia. One of the difficulties in North Kalimantan Province that the road did not reach border villages, making transporting commodities within Kalimantan to border villages more difficult and costly. Meanwhile, the nearby city of Tawau offers lower prices and a diverse choice of products, whereas Nunukan people rely heavily on the neighboring country, Malaysia, for domestic necessities [50].

### Study design

The population-based descriptive cross-sectional study was conducted during the COVID-19 pandemic between February and April 2021. Multistage sampling was used to achieve the sample size for this study [51]. First, all villages or districts were identified based on recent malaria cases that officially reported. Houses within the selected villages or districts were then listed using simple random selection. One family member from each household was selected to represent the family. The inclusion criteria for this study included individuals aged over 15 years who lived in the study areas and had a history of visiting malaria-endemic regions. The exclusion criteria include respondents' residency for less than 6 months. Sample size calculation was based on the Isaac and Michael formula, using the populations of the three provinces [51].

The research tool employed in this study was adapted from a questionnaire utilized by Munajat et al. [52], which included questions on demographic characteristics (age, gender, education level, and occupation), knowledge of malaria (cause, symptoms such as fever and shivering, and prevention), and practices towards malaria illness, malaria prevention practices (such as the use of bed nets and insecticides), and attitudes towards contributing factors of zoonotic malaria transmission. The closed-ended questionnaire was interviewer-guided and was administered in the local language.

### Data and statistical analyses

The data collected were tabulated into an Excel spreadsheet (Microsoft, USA). Data analysis was performed using SPSS (Statistical Package for Social Sciences) version 29 (SPSS, Inc, Chicago, USA). Descriptive data were reported as frequencies and percentages for categorical variables and mean standard deviation (SD) for continuous variables. Chi-squared test was performed to examine the attitudes towards the contributing factors to zoonotic malaria transmission in the six border regencies. The effect of predictive variables (gender, age group, education level and occupation) on good knowledge and good practices was tested using logistic regression. The level of significance was set at *P* < 0.05.

The respondent’s knowledge of malaria was assessed using three items: correct identification of malaria parasite transmission, identification of malaria symptoms, and knowledge of malaria prevention. The knowledge score was calculated by adding responses to all three items [53]. Only those who recognized mosquito bites as the source of malaria received a score of one. For knowledge of malaria symptoms, those who correctly identified malaria symptoms (fever, headache, shivering or chills, sweat, nausea, vomiting and diarrhea) received one mark for each accurate response. For knowledge of malaria prevention measures, respondents who said they slept under bed nets, burned coils, used insecticide spray or repellent, wore protective clothing, took medication and avoided going out in the evening received one mark for each response. The median of the overall score was used as a cutoff to categorize knowledge of malaria into two levels: good (equal to above the median) and poor (below the median) levels of knowledge of malaria [53, 54].

The respondent’s level of practice regarding malaria prevention was assessed based on their ownership and usage of long-lasting insecticide nets (LLINs). Respondents who owned LLINs and used them three or more times per week were considered to have good prevention practices, while those who did not possess LLINs or those who possessed them but used them less than three nights per week were considered to have poor prevention practices [53, 55–58].

## Results

A total of 639 respondents from six regencies in Kalimantan participated in this study (Table 1), with a median age of 37 years. While most respondents were women (53.1%), there were more men participants from the East and North Kalimantan provinces. The majority of respondents had primary education (47.6%), and engaging in the agricultural sector (49.1%) was the most common type of occupation. Overall, the variations were observed across six regencies, with gender distribution, educational background, and occupation differing significantly.

**Table 1:** Demographic data of respondents and malaria control measures in six regencies of Kalimantan border, Indonesia

Characteristic	Overall	Sanggau	Sintang	Kapuas Hulu	Mahakam Ulu	Malinau	Nunukan
Total number of respondents, *n* (%)	639	174	93	143	81	74	74
Gender, *n* (%)							
Women	339 (53.1)	137 (78.7)	52 (55.9)	79 (55.2)	29 (35.8)	24 (32.4)	18 (24.3)
Men	300 (46.9)	37 (21.3)	41 (44.1)	64 (44.8)	52 (64.2)	50 (67.6)	56 (75.7)
Age group, *n* (%)							
Youth (15–24)	87 (13.6)	20 (11.5)	15 (16.1)	25 (17.5)	12 (14.8)	5 (6.8)	10 (13.5)
Adult (25–64)	497 (77.8)	130 (74.7)	71 (76.3)	104 (72.7)	65 (80.2)	65 (87.8)	62 (83.8)
Elderly (≥ 65)	55 (8.6)	24 (13.8)	7 (7.5)	14 (9.8)	4 (4.9)	4 (5.4)	2 (2.7)
Education, *n* (%)							
No formal Education	114 (17.8)	68 (39.1)	10 (10.8)	18 (12.6)	5 (6.2)	8 (10.8)	5 (6.8)
Primary Education	304 (47.6)	76 (43.7)	30 (32.3)	69 (48.3)	45 (55.6)	27 (36.5)	57 (77)
Secondary Education	158 (24.7)	26 (14.9)	32 (34.4)	43 (30.1)	23 (28.4)	23 (31.1)	11 (14.9)
Tertiary Education	63 (9.9)	4 (2.3)	21 (22.6)	13 (9.1)	8 (9.9)	16 (21.6)	1 (1.4)
Occupation, *n* (%)							
Agricultural sector	314 (49.1)	72 (41.4)	25 (26.9)	73 (51)	66 (81.5)	20 (27)	58 (78.4)
Housewife	176 (27.5)	81 (46.6)	32 (34.4)	38 (26.6)	7 (8.6)	9 (12.2)	9 (12.2)
Private employee	51 (8)	7 (4)	17 (18.3)	18 (12.6)	1 (1.2)	6 (8.1)	2 (2.7)
Civil Servant	25 (3.9)	3 (1.7)	4 (4.3)	4 (2.8)	3 (3.7)	11 (14.9)	0 (0)
Student	9 (1.4)	2 (1.1)	3 (3.2)	4 (2.8)	0 (0)	0 (0)	0 (0)
Others	64 (10)	9 (5.2)	12 (12.9)	6 (4.2)	4 (4.9)	28 (37.8)	5 (6.8)
LLIN utilisation/week, n (%)							
Every night	315 (49.3)	112 (64.4)	45 (48.4)	82 (57.3)	34 (42)	22 (29.7)	20 (27)
> 5 nights	2 (0.3)	0 (0)	1 (1.1)	1 (0.7)	0 (0)	0 (0)	0 (0)
3–5 nights	9 (1.4)	7 (4)	1(1.1)	0 (0)	0 (0)	1 (1.4)	0 (0)
< 3 nights	17 (2.7)	7 (4)	3 (3.2)	6 (4.2)	0 (0)	0 (0)	1 (1.4)
Without LLIN	296 (46.3)	48 (27.6)	43 (46.2)	54 (37.8)	47 (58)	51 (68.9)	53 (71.6)
Aware if the bed nets are treated^*^							
No	256 (74.6)	87 (69)	34 (68)	71 (79.8)	31 (91.2)	16 69.6)	17 (81)
Yes	87 (25.4)	39 (31)	16 (32)	18 (20.2)	3 (8.8)	7 (30.4)	4 (19)
IRS within the last 12 months, *n* (%)							
No	579 (90.6)	167 (96)	85 (91.4)	137 (95.8)	63 (77.8)	57 (77)	70 (94.6)
Yes	60 (9.4)	7 (4)	8 (8.6)	6 (4.2)	18 (22.2)	17 (23)	4 (5.4)

^*^Result from LLINs owner

### Malaria awareness knowledge and preventive measure practices

Malaria awareness was tested by determining the depth of the basic knowledge of malaria and preventive measure practice (Table 2). In general, mosquito bites were the most recognized cause of malaria, with an overall percentage of 78.6%. Respondents from Kapuas Hulu Regency had the highest knowledge of mosquito bites as the cause (92.3%). Other potential risk factors, such as dirty surroundings, stagnant water, and poor nutrition, were less commonly identified as causes of malaria.

**Table 2:** Knowledge on cause of malaria, symptoms, prevention methods and care-seeking behaviour among communities in six regencies along the Kalimantan border, Indonesia

Characteristic	Overall	Sanggau	Sintang		Kapuas Hulu	Mahakam Ulu	Malinau	Nunukan
Total number of respondents, n (%)	639	174		93	143	81	74	74
Know the cause of malaria, n (%)								
Mosquito bite	502 (78.6)	93 (53.5)	87 (93.5)		132 (92.3)	75 (92.6)	62 (83.8)	53 (71.6)
Dirty surrounding	91 (14.2)	22 (12.6)	25 (26.9)		17 (11.9)	13 (16)	9 (12.2)	5 (6.8)
Stagnant water	50 (7.8)	14 (8)	11 (11.8)		9 (6.3)	6 (7.4)	6 (8.1)	4 (5.4)
Other insects	19 (2.9)	2 (1.2)	7 (7.5)		4 (2.8)	5 (6.2)	1 (1.4)	0 (0)
Poor nutrition	14 (2.2)	7 (4)	3 (3.2)		1 (0.7)	2 (2.5)	1 (1.4)	0 (0)
No answer	48 (7.5)	36 (20.7)	0 (0)		0 (0)	0 (0)	0 (0)	12 (16.2)
Symptoms of malaria^a^, n (%)								
Fever*	539 (84.3)	95 (54.6)	90 (96.8)		139 (97.2)	76 (93.8)	70 (94.6)	69 (93.2)
Shivering/Chills*	331 (51.8)	66 (37.9)	55 (59.1)		88 (61.5)	51 (63)	38 (51.4)	33 (44.6)
Headache*	154 (24.1)	36 (20.7)	43 (46.2)		29 (20.3)	28 (34.6)	12 (16.2)	6 (8.1)
Sweating*	122 (19.1)	25 (14.4)	23 (24.7)		35 (24.5)	25 (30.9)	6 (8.1)	8 (10.8)
Nausea	75 (11.7)	21 (12.1)	10 (10.7)		13 (9)	10 (12.3)	9 (12.2)	12 (16.2)
Body ache*	52 (8.1)	13 (7.5)	16 (17.2)		8 (5.6)	10 (12.3)	4 (5.4)	1 (1.4)
Vomit	34 (5.3)	14 (8)	7 (7.5)		4 (2.8)	8 (9.9)	1 (1.4)	0 (0)
Anaemia/pale	38 (5.9)	15 (8.6)	9 (9.6)		2 (1.4)	10 (12.3)	2 (2.7)	0 (0)
Convulsion	25 (3.9)	11 (6.3)	7 (7.5)		0 (0)	7 (8.6)	0 (0)	0 (0)
Coma	19 (2.9)	8 (4.6)	6 (6.5)		0 (0)	5 (6.2)	0 (0)	0 (0)
Diarrhea	13 (2)	6 (3.4)	3 (3.2)		0 (0)	4 (4.9)	0 (0)	0 (0)
Know classic symptoms of malaria, n (%)								
5 of 5	16 (2.5)	5 (2.9)	5 (5.4)		1 (0.7)	5 (6.2)	0 (0)	0 (0)
4 of 5	24 (3.8)	8 (4.6)	7 (7.5)		2 (1.4)	6 (7.4)	1 (1.4)	0 (0)
3 of 5	134 (21)	17 (9.8)	28 (30.1)		42 (29.4)	24 (29.6)	13 (17.6)	10 (13.5)
2 of 5	202 (31.6)	32 (18.4)	30 (32.2)		60 (41.9)	25 (30.9)	28 (37.8)	27 (36.5)
1 of 5	198 (31)	64 (36.8)	21 (22.6)		36 (25.2)	17 (21)	30 (40.5)	30 (40.5)
None	65 (10.1)	48 (27.6)	2 (2.2)		2 (1.4)	4 (4.9)	2 (2.7)	7 (9.5)
Prevention of malaria^a^, n (%)								
Sleep under a bed net	425 (66.5)	60 (34.5)	66 (71)		114 (79.7)	66 (81.5)	64 (86.5)	55 (74.3)
Burn coils	262 (41)	68 (39.1)	55 (59)		55 (38.5)	36 (44.4)	25 (33.8)	23 (31.1)
Spray home/surrounding	245 (38.3)	52 (30)	35 (37.6)		87 (60.8)	24 (29.6)	28 (37.8)	19 (25.7)
Drain stagnant water	115 (18)	25 (14.4)	22 (23.7)		35 (24.5)	17 (21)	12 (16.2)	4 (5.4)
Larvicide on water	97 (15.2)	14 (8)	20 (21.5)		29 (20.3)	7 (8.6)	28 (37.8)	9 (12.2)
Wear insect repellent	57 (8.9)	16 (9.2)	21 (22.6)		10 (7)	4 (4.9)	6 (8.1)	0 (0)
Wear protective clothing	43 (6.7)	12 (6.9)	22 (23.6)		2 (1.4)	5 (6.2)	2 (2.7)	0 (0)
Avoid mosquito biting times (evening)	41 (6.4)	14 (8)	11 (11.8)		3 (2.1)	3 (3.7)	5 (6.8)	5 (6.8)
Take medication	25 (3.9)	8 (4.6)	12 (12.9)		3 (2.1)	1 (1.2)	1 (1.4)	0 (0)
Practice on managing malaria illness^a^, n (%)								
Go to the clinic immediately	540 (84.5)	135 (77.6)	77 (82.8)		130 (90.9)	68 (84)	67 (90.5)	63 (85.1)
Purchase medication from a local shop	105 (16.4)	20 (11.5)	27 (29)		12 (8.4)	29 (35.8)	6 (8.1)	11 (14.8)
Seeking treatment from a traditional healer	42 (6.5)	14 (8)	4 (4.3)		11 (7.7)	1 (1.2)	7 (9.5)	5 (6.8)
Wait out the symptoms until well	12 (1.9)	10 (5.7)	2 (2.2)		0 (0)	0 (0)	0 (0)	0 (0)

^a^Some respondents with a combination of more than one answer, *Classic symptoms of malaria

The classic symptoms of malaria were the most widely recognized, which include fever (84.3%), shivering or chills (51.8%), and headache (24.1%) (Table 2). Most of the respondents in each  regency were able to correctly associate fever with malaria, except those from Sanggau regency (54.6%). Other symptoms such as sweating, vomiting, and diarrhea were less commonly identified (overall < 20%). In addition, most respondents could relate to only one (31%) or two (31.6%) classic symptoms (fever and shivering or fever and sweating) of malaria, while only a small proportion (2.5%) knew all five classic symptoms (fever, shivering/chill, sweating, headache and body ache).

Malaria preventive measures were assessed among the respondents. A slight majority owned LLINs (53.7%), and most stated using them every night (49.3%) (Table 1). The proportion of LLIN usage varied across regencies, with West Kalimantan Province (Sanggau, Sintang, and Kapuas Hulu regencies) demonstrating higher nightly usage. Indoor residual spraying (IRS) coverage by each district vector control was limited, with only 9.4% of surveyed households reporting IRS in the past 12 months. Malinau Regency had the highest proportion of IRS responses in the past 12 months (23%). It was observed that sleeping under a bed net (66.5%) and burning mosquito coils (41%) were the most common practices (Table 2). Respondents from Melinau Regency had the highest awareness knowledge of using bed nets (86.5%, *P* < 0.001). Wearing protective clothing and avoiding mosquito biting times with insecticide or repellent were less commonly practiced prevention measures.

In addition, the majority (84.5%) of the respondents sought medical treatment at the clinic for managing malaria. Only a smaller proportion sought treatment from traditional healers (6.5%) or directly purchased medication from local shops (16.4%), such as paracetamol or antimalarial drugs (i.e., hydroxychloroquine sulfate, doxycycline, sulfadoxine–pyrimethamine, and quinine).

### Factors contributing to potential zoonotic malaria parasite transmission

Overall, 64.2% of respondents declared they slept at home every night (Table 3). Nunukan Regency had a significantly higher proportion of respondents spending the night at home every night, compared to other regencies (89.2%, *P* < 0.001). The majority of respondents (63.8%) resided more than 500 m away from the forest. However, most respondents in Sintang Regency lived less than 500 m away (55.9%, *P* < 0.001). Among the respondents (15.8%) who reported encountering monkeys nearby, Sintang Regency had a relatively lower proportion (9.7%, *P* < 0.005) of respondents encountering monkeys within 500 m of their house. Although most respondents lived further from the forest, significantly high proportions (*P* < 0.001) spent nights in the forest, particularly in Sintang, Kapuas Hulu and Mahakam Ulu, due to agricultural work.

**Table 3:** Contributing factors to zoonotic malaria transmission among communities in six regencies along the Kalimantan border, Indonesia

Characteristic	Overall	Sanggau	Sintang	Kapuas Hulu	Mahakam Ulu	Malinau	Nunukan	*P* value
Frequency of spending the night at home, *n* (%)								
Every night	410 (64.2)	124 (71.3)	56 (60.2)	80 (55.9)	44 (54.3)	40 (54.1)	66 (89.2)	< 0.001
Occasionally	229 (35.8)	50 (28.7)	37 (39.8)	63 (44.1)	37 (45.7)	34 (45.9)	8 (10.8)	
Live within 100–500 m from the forest, *n* (%)								
Yes	231 (36.2)	75 (43.1)	52 (55.9)	67 (46.9)	25 (30.9)	9 (12.2)	3 (4.1)	< 0.001
No	408 (63.8)	99 (56.9)	41 (44.1)	76 (53.1)	56 (69.1)	65 (87.8)	71 (95.9)	
Monkey presence within 500 m from the house, *n* (%)								
Yes	101 (15.8)	30 (17.2)	9 (9.7)	27 (18.9)	19 (23.5)	13 (17.6)	3 (4.1)	0.005
No	538 (84.2)	144 (82.8)	84 (90.3)	116 (81.1)	62 (76.5)	61 (82.4)	71 (95.9)	
Spend the night within 500 m of the forest or in the forest, *n* (%)								
Yes	225 (35.2)	50 (28.7)	42 (45.2)	65 (45.5)	36 (44.4)	25 (33.8)	7 (9.5)	< 0.001
No	414 (64.8)	124 (71.3)	51 (54.8)	78 (54.5)	45 (55.6)	49 (66.2)	67(90.5)	

### Factors associated with knowledge and practice on malaria

The univariate and multivariate logistic regression analyses of factors associated with knowledge and practice regarding malaria among communities in six regencies along the Kalimantan border are presented in Tables 4 and 5, respectively. The final model in multivariate logistic regression showed that only education level was significantly associated with good knowledge, including primary education (adjusted odds ratio [aOR] = 3.46; 95% CI 2.13–5.16; P < 0.001), secondary education (aOR = 4.47; 95% CI 2.56–7.82; *P* < 0.001), and tertiary education (aOR = 7.08; 95% CI 3.38–14.85; *P* < 0.001) (Table 4). Regarding practice, several variables were associated with significantly higher odds of good practice regarding malaria in the final model, including women (aOR = 2.25; 95% CI 1.59–3.15; *P* < 0.001), age between 25 and 64 years (aOR = 2.64; 95% CI 1.59–4.36; *P* < 0.001), and age over 65 years (aOR = 3.06; 95% CI 1.43–6.52; *P* = 0.004). Conversely, lower odds were observed among individuals with unspecific occupations (aOR = 0.44; 95% CI 0.24–0.82; *P* = 0.009) (Table 5).

**Table 4:** Univariate and multivariate analyses of respondent’s level of knowledge among communities in six regencies across the Kalimantan border, Indonesia

Category	Good knowledge	Poor knowledge	cOR (95% CI)	*P* value	aOR (95% CI)	*P* value
Overall, *n* (%)	372 (58.2)	267 (41.8)				
Gender, *n* (%)						
Men	180 (60)	120 (40)	1		1	
Women	192 (56.6)	147 (43.4)	0.87 (0.64–1.19)	0.390	1.01 (0.72–1.43)	0.944
Age group, *n* (%)						
Youth (15–24)	53 (60.9)	34 (39.1)	1		1	
Adult (25–64)	295 (59.4)	202 (40.6)	0.94 (0.59–1.49)	0.784	1.01 (0.62–1.62)	0.982
Elderly (≥ 65)	24 (43.6)	31 (56.4)	0.49 (0.25–0.99)	0.045	0.81 (0.39–1.67)	0.559
Education level, *n* (%)						
No formal education	36 (31.6)	78 (68.4)	1		1	
Primary education	185 (60.9)	119 (39.1)	3.37 (2.13–5.32)	< 0.001	3.46 (2.13–5.61)	< 0.001
Secondary education	104(65.8)	54 (34.2)	4.17 (2.49–6.98)	< 0.001	4.47 (2.56–7.82)	< 0.001
Tertiary education	47 (74.6)	16 (25.4)	6.37 (3.19–12.71)	< 0.001	7.08 (3.38–14.85)	< 0.001
Occupation, *n* (%)						
Agricultural sector	180 (57.3)	134 (42.7)	1		1	
Housewife	95 (54)	81 (46)	0.87 (0.60–1.27)	0.474	0.68 (0.43–1.08)	0.102
Private employee	38 (74.5)	13 (25.5)	2.18 (1.12–4.25)	0.023	1.13 (0.55–2.32)	0.734
Civil Servant	16 (64)	9 (36)	1.32 (0.57–3.09)	0.517	0.41 (0.16–1.09)	0.074
Student	3 (33.3)	6 (66.7)	0.37 (0.09–1.52)	0.168	0.25 (0.06–1.07)	0.062
Others	40 (62.5)	24 (37.5)	1.24 (0.71–2.16)	0.445	0.89 (0.49–1.61)	0.699

*cOR* crude odd ratio, *aOR* adjusted odd ratio, *CI* confidence interval

**Table 5:** Univariate and multivariate analyses of respondent’s level of practice on malaria among communities in six regencies across the Kalimantan border, Indonesia

Category	Good practice	Poor practice	cOR (95% CI)	*P* value	aOR (95% CI)	*P* value
Overall, *n* (%)	326 (51)	313 (49)				
Gender, *n* (%)						
Men	118 (39.3)	182 (60.7)	1		1	
Women	208 (61.4)	131 (38.6)	2.45 (1.78–3.37)	< 0.001	2.25 (1.59–3.15)	< 0.001
Age group, *n* (%)						
Youth (15–24)	29 (33.3)	58 (66.7)	1		1	
Adult (25–64)	266 (53.5)	231 (46.5)	2.31 (1.43–3.72)	0.001	2.64 (1.59–4.36)	< 0.001
Elderly (≥ 65)	31 (56.4)	24 (43.6)	2.58 (1.29–5.17)	0.007	3.06 (1.43–6.52)	0.004
Education level, *n* (%)						
No formal education	73 (64)	41 (36)	1		1	
Primary education	151 (49.7)	153 (50.3)	0.55 (0.36–0.86)	0.009	0.75 (0.46–1.21)	0.245
Secondary education	71 (44.9)	87 (55.1)	0.46 (0.28–0.75)	0.002	0.58 (0.33–1.01)	0.052
Tertiary education	31 (49.2)	32 (50.8)	0.54 (0.29–1.02)	0.056	0.56 (0.28–1.11)	0.096
Occupation, *n* (%)						
Agricultural sector	153 (48.7)	161 (51.3)	1		1	
Housewife	112 (63.6)	64 (36.4)	1.84 (1.26–2.69)	0.002	1.31 (0.81–2.08)	0.271
Private employee	27 (52.9)	24 (47.1)	1.18 (0.65–2.14)	0.577	1.68 (0.87–3.22)	0.121
Civil Servant	11 (44)	14 (56)	0.83 (0.36–1.88)	0.649	0.95 (0.37–2.46)	0.918
Student	3 (33.3)	6 (66.7)	0.53 (0.13–2.14)	0.371	0.92 (0.21–4.14)	0.913
Others	20 (31.3)	44 (68.7)	0.48 (0.27–0.85)	0.012	0.44 (0.24–0.82)	0.009

*cOR* crude odd ratio, *aOR* adjusted odd ratio, *CI* confidence interval

## Discussion

This study was conducted in three Kalimantan provinces bordering MalaysianBorneo in a low-endemicity malaria area with an Annual Parasite Incidence (API) of 0 to 1 [49]. The study sites, however, were geographically close to the Kapit Division in Sarawak, Malaysia, which is a *P. knowlesi*-focused area (Fig. 1). This study describes the demographics, knowledge and practices related to malaria disease prevention among the community in six regencies along the Indonesia–Malaysia border.

More than 70% of respondents in this study were able to associate mosquito bites with malaria, demonstrating their basic understanding of malaria as a vector-borne disease. However, there was still some uncertainty about the aetiology of malaria, with respondents attributing the disease to other factors, such as other insects (flies, fleas, lice, or cockroaches), dirty surroundings, poor nutrition and *Aedes* mosquitoes. Respondents identified disease-causing mosquitoes as *Aedes* mosquitoes, because they were unaware of *Anopheles*' role as a malaria vector. Dengue fever cases fluctuated in 2019–2023 in almost every island in Indonesia [49], prompting an extensive dengue vector prevention programme, such as prevention commercials in social media and introduction of larva observer (*Jumantik/juru pemantau jentik*) in the community, as well as the widespread recognition of the *Aedes* mosquito. Moreover, the Ministry of Health has introduced an acronym for dengue vector prevention which is 3 M *Plus*: *Menutup* (close all types of containers that could store water), *Membersihkan* (clean large containers in the bathroom), *Mendaur ulang* (recycle waste) and *Plus* (additional measures, such as the use of larvicide, repellent, or insecticide) [49, 59]. Furthermore, during this study, the description of dengue preventive measures was more commonly mentioned by respondents than those for malaria. The campaign against malaria has mostly focused on the highly endemic area in the eastern part of Indonesia, such as in the Papua Province, where annual parasitic incidence is > 5 [60, 61]. Although the respondents were well-versed in the cause and some symptoms of malaria (such as fever, shivering and sweating), some require more information on malaria prevention and the potential risk of zoonotic malaria infection. The practice of using LLINs or wearing protective clothing in the evening for malaria prevention was not common in every district. To ensure that the correct message is delivered to the communities, local healthcare personnel need to constantly recall the information. Furthermore, continued malaria surveillance, including active case detection by healthcare personnel through house-to-house visits and migration surveillance, is crucial and must be continuously emphasized to efficiently detect, treat, and reduce malaria. A study on malaria prevention practice in rural East Nusa Tenggara Province and Java Island showed a significant relationship between education and malaria knowledge, with adult and secondary education level respondents having higher knowledge than those with primary and no education [62, 63]. In contrast, studies in Northwest Ethiopia and Ghana found no significant association between sociodemographic factors (such as age, education, gender, occupation, and religion) and malaria prevention practices [64, 65]. Another study in the Democratic Republic of Congo highlighted the importance of education in increasing the use of LLINs, showing that women and children in school were more likely to use them [66]. Similarly, in Tanzania, pupils demonstrated a strong awareness of malaria [67]. Although a study in Guinea showed that there was no association between gender or level of education and malaria knowledge, respondents who had received free bed nets during national campaigns, more than half of them slept under one [68].

Health education on malaria prevention can be delivered in schools, allowing students to share their knowledge at home. In Indonesia’s school curriculum, disease prevention and sanitation education are taught in both primary and secondary schools. Given that most respondents completed primary and secondary education, their educational background likely contributed to their high level of malaria prevention knowledge. This positive perception of malaria prevention should be strengthened by government support, such as continued malaria preventive promotion in schools or via television, social media and radio, as indicated in the dengue prevention programme, free bed net distribution, and active case detection.

Long-lasting insecticide-treated bed nets (LLINs) have been introduced through subnational campaigns in endemic regions in Indonesia since 2005 [69, 70]. More than half of the respondents in this study owned bed nets and declared to use them regularly. The LLINs were given for free and distributed by the government to communities in high (API > 5), moderate (API 1–5), or low (API < 1) endemic areas, during antenatal visits, outbreaks, and natural disasters [61, 70]. Since the study sites were not highly endemic, LLINs were distributed only based on reported malaria cases. As a result, when the given net wore out, people left to obtain their own, which was an untreated bed net that circulated in the market. The LLINs usage is contingent on net access [71]. The use of LLINs can significantly reduce malaria incidence rate [72], highlighting the importance of encouraging continued ownership and regular uses of LLINs through ongoing government support. As reported by the health officer in each district during this study, vector control intervention was performed based on reported mosquito-borne disease, such as dengue or malaria. IRS was only done in high-risk villages (API > 5), villages with low coverage of LLINs (< 80%), or during malaria disease outbreaks [61, 73]. Since the number of reported malaria cases was low in each regency, IRS activities were relatively infrequent. Fogging was frequently done in every district, since the district health office responded with fogging to all reported dengue cases in the area. Fogging carried out to target adult mosquitoes such *Aedes* mosquitoes (vectors of dengue, chikungunya, zika), *Culex* mosquitoes (vectors of Japanese Encephalitis), and *Anopheles* mosquitoes (vectors of malaria). Fogging for malaria by the Indonesian Health Authority will be done if malaria cases are still reported 3 months following IRS and LLINs distribution, and a malaria outbreak [74].

In Malaysia, LLINs and IRS have been integral to the national malaria elimination strategy [75, 76]. Both Indonesia and Malaysia have viable malaria elimination programmes, but high coverage of vector control efforts, political stability, and government support must be assured. Long-term financial planning for elimination and retention programmes is critical, without sustained financial support, all investment and effort would potentially be squandered. There should be efforts to improve ownership of bed nets by continuous mass-free distribution supported with adequate behaviour change intervention, durability monitoring of bed nets and vector pesticide resistance tracking in the border area.

In general, respondents in the present study demonstrated a positive attitude towards seeking treatment at health facilities when suspecting malaria. The accessibility to nearby facilities and availability of free medical treatments provided by the government have been an advantage in reducing malaria cases across the regions. While modern medical treatment was a primary choice, traditional alternative treatments or rituals were still preferred, especially in the West and East Kalimantan provinces. Some people believe that diseases arise from the supernatural outside of human consciousness can only be cured by magical means [77]. Interestingly, the shaman does not use processed herbal materials to treat the disease, but rather ingredients such as eggs, betel leaves, gambier (*Uncaria gambir*), areca nuts, nails, rice, and kitchen ash due to disturbances from spirits. A similar behaviour was observed among the aboriginal communities in Peninsular Malaysia, where malaria is associated with ghosts and evil spirits [52, 78]. People in Nigeria, on the other hand, believe that severe malaria symptoms, particularly convulsions, come from within and are inherited from their parents, and they believe in the efficacy of their herbal remedies [79]. In addition, self-medication, such as purchasing medicine (e.g., paracetamol, chloroquine, or other antimalarial drugs) at the local shops or pharmacies, was a common practice. However, this practice should not be encouraged, as it poses issues related to the sale of counterfeit antimalarial drugs [80, 81], which could potentially increase the spread of drug-resistant parasites [82].

A recent preliminary molecular surveillance study conducted in West Kalimantan Province has highlighted the presence for zoonotic malaria among asymptomatic communities in the Kapuas Hulu Regency [83]. This finding is strongly supported by this present study, which indicates that respondents living near forests or spending nights in forested areas may have encountered monkeys in their vicinity. Higher fragmentation of oil palm plantations was associated with increased *P. knowlesi* exposure, implying that changes in habitat configuration and fragmented landscapes may promote interaction between populations at habitat edges that could be risk to humans [41]. Working on plantations, farming occupation, clearing vegetation, and having long grass around the house have been linked to *P. knowlesi* risk in Sabah as well as throughout Southeast Asia [84, 85]. In West Kalimantan, most respondents spent nights in the forest for activities, such as farming and gathering forest products, while in East Kalimantan, overnight stays were primarily for hunting and collecting agarwood (k*ayu gaharu*). Such forest-related activities potentially increase the likelihood of contracting *P. knowlesi* from deep-forest-dwelling macaques [86].

Moreover, the diversity of mosquito vectors for simian *Plasmodium* species is noteworthy, including the Dirus Complex and Leucosphyrus Complex [43, 87]. The Dirus Complex (*An. dirus* and *An. cracens*) has been found in various environments, such as woodland, forest edges, and settlement areas, while the Leucosphyrus Complex (*An. latens, An. balabacensis* and *An. introlatus*) is typically found in disturbed forests, where natural forests have been modified by human activities into plantations or logging concessions [44]. The possibility of zoonotic malaria infection may increase when monkey habitats are disturbed, bringing both vectors and humans into contact, which need to be explored further. Given that most respondents work in agricultural settings (forest, plantation, and farming), the risk of acquiring zoonotic malaria is inevitable. Data on macaques and vectors in this study area are scarce, and the significance of these agroforestry systems should be investigated further.

Indeed, Malaysia is dealing with zoonosis malaria by *P. knowlesi* infection as a major health problem [88], with high cases that are unparallel with Kalimantan, although three Indonesian provinces share a border with Sarawak and Sabah states of Malaysian Borneo. The macaque natural hosts (*M. fascicularis* and *M. nemestrina*) of *P. knowlesi* [44, 86] and mosquito vectors (*Anopheles leucosphyrus*) [89] are found on both sides of the border, hence the risk of knowlesi malaria is similar in Kalimantan and Malaysian Borneo. Even though both countries faced the same risk, there was no specific cross-border collaboration on malaria surveillance and control.

Cross-border collaboration between Indonesia and neighbouring countries such as Malaysia, Timor Leste, and Papua New Guinea is an important component in eliminating malaria disease. Cross-border collaboration has been established between the Ministries of Health of both Indonesia and Timor Leste, with an agreement period of 2022–2026 [90]. The cross-border action plan addresses malaria examination and treatment, health training, maternal and child health services, immunisation and nutrition. Therefore, similar collaboration could be done with Malaysia, particularly within Borneo Island [90].

A stronger collaboration among ministries, particularly the Ministries of Health, Forestry and Environment, and Home Affairs, as well as the WHO, is essential to sustain malaria elimination programmes and meet the malaria elimination target for all Indonesian islands by 2030. More government collaborations across provinces, regencies, and cities in Indonesia are required to anticipate the risk of malaria and zoonosis malaria.

This study has certain limitations that should be noted. The inclusion of all men household members should improve the accuracy of the finding, as men are over-represented in agricultural and forest-related activities, increasing their likelihood of zoonotic malaria exposure [84–86]. A more representative sample of male participants would enable a better understanding of occupational risk variables. Furthermore, the use of self-reported questionnaires may include recall bias and social desirability bias, in which individuals incorrectly recall previous exposures or provide replies that they believe are more acceptable. This could result in misclassification of risk variables and reduce data accuracy. Some questions, involving the administration and distribution of LLINs, unprescribed antimalarial use in the community, herbal remedies usage by traditional healer and vector-related issues, should also be expanded and studied further. Future studies may employ objective data collection methods, such as field observations, focus-group discussions with high-risk communities (such as those who work in the forest, mine worker or soldier), or GPS monitoring for each one, to confirm self-reported data and increase dependability. Resolving these limitations will potentially enhance our understanding of the risks of zoonotic malaria transmission in these regions.

## Conclusion

This study found that these communities have exceptional level of malaria knowledge, practices regarding malaria, and attitudes towards zoonotic malaria. Deforestation, land use changes, and enormous rainforest degradation in Kalimantan are gravely endangering the local ecosystem and raising the risk of *P. knowlesi* and zoonotic malaria infections for locals. The emergence of zoonotic malaria in Indonesia has surely altered the dynamics of malaria management and control in the pursuit of total elimination from the human population. However, it is essential to emphasise the significance of continuous malaria surveillance within this community, as it is essential for maintaining the current low malaria cases and achieving the goal of malaria-free status in the country by 2030.

## Data Availability

No datasets were generated or analysed during the current study.
